# Impact of enhanced recovery after surgery on postoperative rehabilitation, inflammation, and immunity in gastric carcinoma patients: a randomized clinical trial

**DOI:** 10.1590/1414-431X20198265

**Published:** 2019-05-16

**Authors:** Wu-Ke Wang, Chao-Yong Tu, Chu-Xiao Shao, Wei Chen, Qing-Yun Zhou, Jing-De Zhu, Hong-Tao Xu

**Affiliations:** 1 Department of General Surgery, Hwa Mei Hospital, University of Chinese Academy of Sciences, Ningbo, Zhejiang, China; 2 Department of General Surgery, Ningbo No. 2 Hospital of Zhejiang University, Ningbo, Zhejiang, China; 3 Department of General Surgery, The Fifth Affiliated Hospital of Wenzhou Medical University, Lishui, Zhejiang, China; 4 Department of General Surgery, Lishui Hospital, Zhejiang University School of Medicine, Lishui, Zhejiang, China; 5 Cancer Institute of Integrated Traditional Chinese and Western Medicine, Key Laboratory of Cancer Prevention and Therapy Combining Traditional Chinese and Western Medicine, Zhejiang Academy of Traditional Chinese Medicine, Hangzhou, Zhejiang, China; 6 Department of Medical Oncology, Tongde Hospital of Zhejiang Province, Hangzhou, Zhejiang, China

**Keywords:** Enhanced recovery after surgery, Perioperative period, Gastric carcinoma, Inflammatory response, Immune function

## Abstract

We determined the effects of enhanced recovery after surgery (ERAS) in patients undergoing radical surgery for gastric carcinoma. Sixty patients undergoing radical gastrectomy for gastric carcinoma in Lishui Hospital between March and October 2016 were randomized to receive either ERAS (30 patients) or conventional care (30 patients, controls). Clinical, economic, and laboratory indices were analyzed. ERAS patients showed faster recovery and shorter postoperative hospital stays than the controls (P<0.05). Some clinical indices (i.e., time to first flatus and defecation, time to removal of drainage tubes, time to resumption of oral feeding, time to postoperative mobilization, and postoperative complications) were significantly better in ERAS patients than in controls. Duration of postoperative infusion was lower in ERAS patients than in controls (P<0.05). In ERAS patients, serum albumin and prealbumin were higher on postoperative day 7, C-reactive protein was lower on postoperative days 3 and 7, and neutrophil count was lower on postoperative day 3 compared to the values in controls (P<0.05 for all). IgM levels were higher in ERAS patients on postoperative days 3 and 7 (P<0.05), while IgG levels were higher on postoperative day 3 (P<0.05). Total T lymphocytes were higher in ERAS patients on postoperative day 3, while helper T cells and CD4^+^/CD8^+^ ratio were higher on postoperative days 3 and 7 (P<0.05 for all). In gastric carcinoma patients, ERAS may reduce perioperative inflammation, improve immunity and postoperative nutrition, shorten hospitalization, and enhance rehabilitation.

## Introduction

Gastric cancer is one of the most common malignancies in the world (1). According to a recent estimate, 952,000 new cases of gastric cancer are diagnosed every year, making it the sixth most common malignancy in the world. Furthermore, gastric cancer leads to 732,000 deaths per year, which makes it the fourth most common cause of cancer-related deaths (2,3). More than 40% of all cases of gastric cancer are in China, where the incidence of this cancer has been increasing in recent years (4).

Surgical treatment remains the only hope for a cure. Open resection is, however, associated with major trauma, numerous complications, slow recovery, prolonged hospitalization, and many other problems (5). Laparoscopic radical gastrectomy is currently the treatment of choice for gastric cancer (6). For advanced gastric cancer, where there is a high risk of recurrence and metastasis, postoperative chemotherapy can improve survival. Because poor recovery after surgery may delay the initiation of chemotherapy, surgeons are increasingly focusing on methods to hasten postoperative recovery.

The concept of enhanced recovery after surgery (ERAS), also known as fast-track surgery, was first proposed by Prof. Henrik Kehlet of Denmark in 1997 (7). ERAS uses evidence-based medical strategies to minimize the effects of the stress associated with surgery. It attempts to restore normal physiological function in the shortest possible time, with the ultimate aim of reducing postoperative complications, shortening hospitalization duration, and promoting rapid recovery (8–10). The ERAS concept does not seek to introduce innovations in gastrectomy, but rather focuses on the optimization of conventional perioperative treatment. ERAS was first applied in cardiothoracic surgery and was largely successful. Over the last 20 years, the principles and methods of ERAS have been greatly improved, with demonstrable success in the fields of general surgery, cardiac surgery, urology, and especially, colorectal surgery. Various guidelines have been published by international bodies for the application of ERAS in different surgical fields, including colorectal surgery (11), pancreaticoduodenal surgery (12), weight-loss surgery, gynecology, and breast surgery. Although the core concept of ERAS remains consistent, some of the measures vary with the type of surgery.

ERAS acts primarily by reducing the stress response during the perioperative period (13). Patients with gastric cancer invariably have some degree of malnutrition and immunological deficiency even before surgery. The strong stress response triggered by surgery and anesthesia further weakens the immune system, resulting in delayed healing and increasing the risk of postoperative infection, intestinal paralysis, anastomotic fistula formation, and other complications (14,15).

Research on ERAS and radical gastrectomy has mostly focused on changes in clinical indicators. Therefore, the present study aimed to determine the safety, feasibility, and clinical efficacy of ERAS in patients undergoing radical gastrectomy and to explore the impact of ERAS on the inflammatory response, immune system, and postoperative recovery.

## Material and Methods

### Ethics and consent

This study was approved by the Ethics Committee of Lishui Hospital, and all procedures were in accordance with the ethical standards laid down in the 1964 Declaration of Helsinki and its later amendments. Informed consent was obtained from the patients and their families before enrollment in the study.

### Patient selection

Gastric cancer patients who underwent radical gastrectomy at the Lishui Hospital between March and October 2016 were selected for this clinical trial. The inclusion criteria were as follows: a) age ≤75 years, b) gastric cancer diagnosed by preoperative gastroscopic biopsy; c) non-emergency surgery, and d) no preoperative radio- or chemotherapy. The exclusion criteria were as follows: a) conditions that made cooperation with the ERAS program impossible (e.g., mental disorder and paralysis), b) severe organ dysfunction, such as heart, brain, and liver dysfunction; c) severe malnutrition, and d) distant metastasis possibly necessitating the resection of other organs.

**Figure 1:**
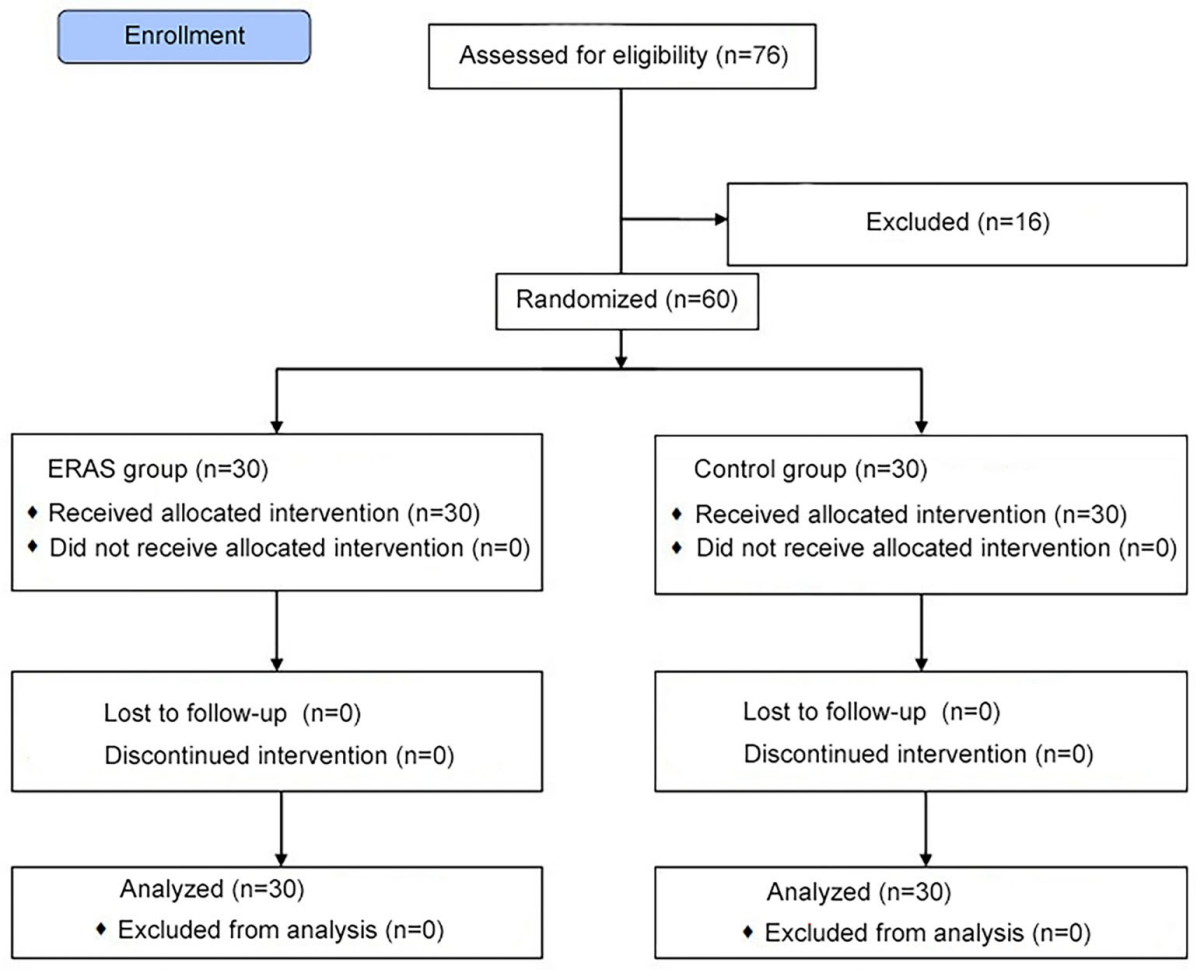
Flow diagram of patient selection and allocation.

Patients could also be dropped from the study after enrollment if any of the following conditions occurred: a) radical resection could not be performed for any reason; b) serious complications requiring rescue measures occurred during or after surgery, and c) the patients or their families requested withdrawal from the study. After enrollment, patients were randomly assigned to the ERAS group or the control group using the sealed envelope method. A flow diagram of patient selection and allocation is shown in Figure 1.

### Perioperative treatment

In the study group, ERAS measures were strictly implemented during the perioperative period. If a certain measure could not be achieved, it was to be postponed or modified as appropriate, and the patient’s physical condition and tolerance were to be reassessed to ensure the implementation of follow-up measures. The ERAS protocol is shown in Table 1.

**Table 1:** Enhanced recovery after surgery (ERAS) protocol.

Time point	Protocol
On admission	1. Preoperative health education 2. No smoking or alcohol 3. Preoperative physical examination to assess nutritional status and rule out contraindications to surgery
Day before surgery	1. No routine bowel preparation (except in case of constipation) 2. Oral administration of 1000 mL of 10% glucose solution at 10 PM on the night before surgery, with another 300 mL at 6 AM the next morning (replaced by saline in patients with diabetes) 3. No solid food 6 h before surgery, and no oral fluids 2 h before surgery 4. Anesthesia consultation, skin preparation, blood examination, insertion of indwelling gastric tube and urinary catheter, and prophylactic antibiotics
Day of surgery	1. General anesthesia alone or in combination with thoracic epidural anesthesia 2. Electric blanket and abdominal-temperature saline irrigation to maintain body temperature during surgery 3. Decision to use abdominal drainage tubes depended on the surgical conditions 4. Intravenous infusion of fluid, calculated as 15–20 mL/kg + volume of blood loss during surgery 5. Subcutaneous infiltration anesthesia + intravenous/epidural analgesic pump anesthesia + intravenous nonsteroidal anti-inflammatory drug (e.g., 50 mg flurbiprofen) + 100 mg tramadol orally 6. Attempt to drink warm water (∼50 mL/h) 6 h after surgery 7. Routine prevention of nausea and vomiting for 2–3 days
Postoperative day 1	1. Early mobilization encouraged 2. Oral fluid intake increased to 500 mL, intravenous fluid volume reduced, total caloric intake limited to 25–30 kcal/kg per day 3. Gastric tube removed according to accepted criteria for extubation^a^ 4. Intermittent urinary catheterization started to train the bladder and stopped when bladder sensation returned to normal 5. Oral lactulose administered for 2 days (in general) and stopped after passing of flatus 6. Chewing gum to stimulate return of normal gastrointestinal function
Postoperative day 2	1. Encouragement to continue and prolong out-of-bed activities 2. Oral fluid intake increased to 1000 mL, liquid diet (such as small amounts of rice soup) started, intravenous fluids reduced so that total intake was unaffected 3. Antibiotics stopped if there was no evidence of infection 4. Gastric tube removed if not obstructed^a^ 5. If urinary catheter was not obstructed, it was removed after completion of bladder training
Postoperative day 3	1. Encouragement to continue and prolong out-of-bed activities 2. Oral fluid intake gradually increased to 1500 mL gradually, amount of liquid diet increased, intravenous fluid volume gradually reduced so that total intake remained the same 3. Abdominal drainage tube removed^b^ after evaluation for 2 days
Postoperative day 4	1. Encouragement to continue and prolong out-of-bed activities 2. Frequent small amounts of oral fluids, small amounts of semi-liquid foods (porridge, noodles, or other soft foods), intravenous fluids stopped if possible, and oral intake increased to maintain the total intake
Postoperative day 5 to discharge	1. Encouragement to continue and prolong out-of-bed activities 2. Frequent small amounts of oral fluids, with gradual transition to total semi-liquid diet and soft foods; amount of total intake amount maintained

^a^The gastric tube could be removed when the amount of drainage was <100 mL/d; the drained fluid was not blood-tinged, and flatus had been passed. ^b^The abdominal drainage tube could be removed when abdominal infection, anastomotic fistula, and other postoperative complications were ruled out, and the drainage volume was <10 mL/d for 2 days.

The control group received conventional perioperative treatment, which included routine preoperative health education, preoperative intestinal preparation with the oral administration of polyethylene glycol-electrolyte powder, fasting for 8 h before surgery, and no water for 6 h before surgery. Postoperatively, out-of-bed activities were arranged according to the will of the patients. The amount of intravenous fluids was not controlled, and oral fluids and food were permitted after flatus was passed. Analgesics were administered only if the patient complained of intolerable pain. The timing of the removal of drainage tubes (i.e., gastric tube, urinary catheter, and abdominal drainage tube) depended on the attending doctor’s judgment.

### Data collection and study endpoints

Gender, age, American Society of Anesthesiologists (ASA) grade, tumor stage, type of gastrectomy, laparoscopic resection, operation time, and intraoperative blood loss were recorded for each patient.

The primary endpoint was the duration of postoperative hospital stay, measured from the day of surgery to the day of discharge. Patients were eligible for discharge when: a) they had no pain or their pain could be controlled with oral analgesics; b) their daily oral intake was sufficient to meet their energy requirements; c) they could perform out-of-bed activities freely, and d) they consented to be discharged and were willing to continue with the ERAS protocol at home.

The secondary outcomes included postoperative clinical, economic, and laboratory indices. The postoperative clinical indices included time to first flatus, time to first defecation, time to removal of drainage tubes (gastric tube, urinary catheter, and abdominal drainage tube), time to resumption of oral feeding (fluids, semi-liquid diet), time to out-of-bed activities, and postoperative complications. The economic indices included the duration of intravenous infusion and hospitalization costs. The laboratory indices included: a) nutritional indices (serum albumin and prealbumin); b) inflammatory indices (C-reactive protein (CRP) and neutrophil count), and c) immunological indices (IgG, IgA, IgM, complements C3 and C4, total T lymphocytes, helper T cells, cytotoxic T cells, and CD4^+^/CD8^+^ ratio).

All laboratory tests were performed by the same person at all time points (before surgery, and on postoperative days 1, 3, and 7). All tests were performed in the clinical laboratory of Lishui Hospital.

### Statistical analysis

Measurement data are reported as means±SD and were compared between groups using Student's *t*-test. Enumeration data are reported as percentages and compared between groups using the chi-squared test or Fisher exact test. Statistical analysis was performed using SPSS v19.0 (IBM Corp., USA). Statistical significance was set at P<0.05 or P<0.01.

## Results

### Baseline characteristics

A total of 76 gastric cancer patients underwent radical gastrectomy in Lishui Hospital during the study period. Of these, 9 patients did not meet the inclusion criteria, and 7 patients declined to participate. Of the remaining 60 gastric cancer patients, 30 were assigned to receive ERAS in the perioperative period (ERAS group) and 30 were assigned to receive conventional care (control group). There were no significant differences between the ERAS and control groups in terms of the mean age, gender ratio, ASA grade, TNM stage, type of gastrectomy, laparoscopic resection, operation time, and intraoperative blood loss (Table 2).

**Table 2:** Comparison of baseline characteristics and surgical conditions between the two groups of patients.

Characteristic	ERAS group	Control group	Test value	P
Gender, M/F	25/5	23/7	χ^2^ = 0.417	0.518
Age in years,	58.22±4.31	59.26±5.35	*t =* 0.829	0.410
ASA grade, n, I/II	10/20	12/18	χ^2^ = 0.287	0.592
Tumor stage, n, I/II/III	7/12/11	5/9/16	χ^2^ = 1.688	0.430
Type of gastrectomy, n			χ^2^ = 0.315	0.854
Proximal	3	4		
Distal	17	15		
Total	10	11		
Open/laparoscopic resection	11/19	13/17	χ^2^ = 0.278	0.598
Operation time, min	187.47±23.26	192.01±31.14	*t =* 0.640	0.525
Intraoperative blood loss, mL	133.33±93.21	156.45±80.02	*t =* 1.031	0.307

Data are reported as means±SD or number. ERAS: enhanced recovery after surgery; ASA: American Society of Anesthesiologists.

### Primary endpoint and clinical indicators

Postoperative hospital stay was significantly shorter in the ERAS patients than in the control patients (8.89±3.27 *vs* 10.76 ± 4.58 days, P=0.039; Table 3). The time to first flatus and defecation, time to removal of drainage tubes, time to resumption of oral feeding, and time to postoperative out-of-bed activities were all significantly shorter in the ERAS patients than in the control patients (Table 3).

**Table 3:** Comparison of postoperative clinical course between the two groups of patients.

Item	ERAS group	Control group	Test value	P
Postoperative hospital stay (days)	8.89±3.27	10.76±4.58	*t* = 2.112	0.039
Time to first flatus, days	2.63±1.07	3.35±1.32	*t* = 2.321	0.024
Time to first defecation, days	4.25±3.02	5.68±2.36	*t* = 2.044	0.045
Time to removal of drainage tubes, days				
Gastric tube	1.56±0.76	3.22±1.75	*t* = 4.766	<0.01
Urinary catheter	1.22±0.99	3.86±1.23	*t* = 9.158	<0.01
Abdominal drainage tube	4.47±1.01	6.46±1.95	*t* = 4.963	<0.01
Time to resumption of oral intake, days				
Time to complete transition to liquid diet	3.82±1.81	5.15±2.25	*t* = 2.523	0.014
Time to complete transition to semi-liquid diet	5.67±2.31	7.21±3.07	*t* = 2.195	0.032
Postoperative mobilization, n			χ^2^ = 15.994	<0.01
Postoperative day 1	15	2		
Postoperative day 3	9	10		
Postoperative complications, n	7	9	χ^2^ = 0.341	0.559
Mild	6	7	χ^2^ = 0.098	0.754
Nausea and vomiting	3	2		
Incision infection	1	2		
Acute urinary retention	2	1		
Urinary tract infection	0	2		
Severe	2	3	χ^2^ = 0.218	0.641
Gastrointestinal stasis	1	1		
Anastomotic fistula	0	0		
Intestinal obstruction	1	2		
Pulmonary infection	0	0		
Deep vein thrombosis	0	0		

Data are reported as means±SD or number. When calculating the total number of postoperative complications, if the same patient had two or more complications, it was only recorded as one person, not to repeat the calculation. ERAS: enhanced recovery after surgery.

A total of 8 postoperative complications (in 7 patients) occurred in the ERAS group: nausea and vomiting (n=3), acute urinary retention (n=2), incision infection (n=1), gastrointestinal stasis (n=1), and intestinal obstruction (n=1). One patient had both gastrointestinal emptying dysfunction and acute urinary retention. In the control group, 10 postoperative complications (in 9 patients) occurred: nausea and vomiting (n=2), urinary tract infection (n=2), incision infection (n=2), intestinal obstruction (n=2), acute urinary retention (n=1), and gastrointestinal emptying dysfunction (n=1). One patient had both intestinal obstruction and incision infection. The rates of both mild and serious complications were lower in the ERAS group than in the control group, although the difference was not statistically significant (Table 3).

### Economic indicators

Postoperative infusion time (6.42 ± 3.22 days *vs* 9.15 ± 3.06 days, P<0.01) and hospital stay (8.89±3.27 *vs* 10.76 ± 4.58 days, P=0.039) were significantly shorter in the ERAS group than in the control group. The total hospitalization cost was also lower in the ERAS group than in the control group, but the difference was not statistically significant (US$4800.99±681.73 *vs* US$5173.89±973.50, P>0.05; Table 4).

**Table 4:** Comparison of economic indicators between two groups of patients.

Item	ERAS group	Control group	Test value	P
Postoperative infusion time (days)	6.42±3.22	9.15±3.06	*t* = 3.366	<0.01
Postoperative hospital stay (days)	8.89±3.27	10.76±4.58	*t* = 2.112	0.039
Hospitalization cost (US$)	4800.99±681.73	5173.89±973.50	*t* = 1.719	0.091

Data are reported as means±SD. ERAS: enhanced recovery after surgery.

### Nutritional indicators

In both groups, the serum albumin and prealbumin levels initially decreased after surgery and then increased. On postoperative days 1 and 3, these levels were higher in the ERAS group than in the control group, but the differences were not statistically significant (P>0.05). On postoperative day 7, however, the difference became statistically significant (P<0.05, Table 5).

**Table 5:** Comparison of nutritional indicators between the two groups.

Item	ERAS group	Control group	Test value	P
Serum albumin, g/L				
At admission	39.13±6.83	38.83±7.30	*t* = 0.164	0.870
Postoperative day 1	32.34±5.27	30.59±5.05	*t* = 1.313	0.194
Postoperative day 3	34.93±6.62	32.28±5.34	*t* = 1.706	0.093
Postoperative day 7	36.91±5.69	34.09±4.83	*t* = 2.069	0.043
Serum prealbumin, mg/L				
At admission	223.54±25.39	227.91±22.36	*t* = 0.707	0.482
Postoperative day 1	183.32±23.98	177.39±21.17	*t* = 1.015	0.314
Postoperative day 3	204.79±24.43	193.66±25.34	*t* = 1.732	0.089
Postoperative day 7	213.99±20.36	203.55±19.42	*t* = 2.032	0.047

Data are reported as means±SD. ERAS: enhanced recovery after surgery.

### Inflammatory indicators

In both groups, the postoperative CRP level initially increased, peaked on postoperative day 3, and then began to decline. On postoperative day 1, the CRP level was lower in the ERAS group than in the control group, but the difference was not statistically significant (P>0.05). On postoperative days 3 and 7, however, the CRP levels were significantly lower in the ERAS group than in the control group (P<0.05).

Neutrophil count initially increased and then decreased. The count was lower in the ERAS group than in the control group on postoperative days 1, 3, and 7, but the difference was statistically significant only on postoperative day 3 (P<0.05, Table 6).

**Table 6:** Comparison of inflammatory indicators between the two groups of patients.

Item	ERAS group	Control group	Test value	P
CRP, mg/L				
At admission	3.36±1.23	3.20±1.51	*t* = 0.450	0.654
Postoperative day 1	59.22±11.38	62.57±12.32	*t =* 1.094	0.278
Postoperative day 3	79.31±19.04	90.23±21.24	*t* = 2.097	0.040
Postoperative day 7	23.14±13.49	32.78±15.35	*t* = 2.584	0.012
Neutrophil, %				
At admission	65.56±18.89	67.32±22.03	*t* = 0.332	0.741
Postoperative day 1	86.36±17.27	89.87±18.54	*t* = 0.759	0.451
Postoperative day 3	76.05±15.96	84.59±16.05	*t* = 2.067	0.043
Postoperative day 7	68.06±14.33	73.25±16.86	*t* = 1.285	0.204

Data are reported as means±SD or number. ERAS: enhanced recovery after surgery; CRP: C-reactive protein.

### Immunoglobulin and complement levels

The postoperative IgG, IgA, and IgM levels initially decreased and then increased in both groups. These levels were generally higher in the ERAS group than in the control group, and the differences between the groups were statistically significant on postoperative days 3 and 7 in the case of the IgG levels (P<0.05) and on postoperative day 3 in the case of the IgM level (P<0.05, Table 7).

**Table 7:** Comparison of immunoglobulin and complement levels between the two groups of patients.

Item	ERAS group	Control group	Test value	P
IgG, g/L				
At admission	11.64±2.68	11.25±3.12	*t* = 0.519	0.605
Postoperative day 3	8.73±2.21	7.12±2.56	*t* = 2.607	0.011
Postoperative day 7	10.23±2.12	8.74±2.33	*t* = 2.037	0.046
IgA, g/L				
At admission	2.38±0.67	2.42±0.88	*t* = 0.198	0.844
Postoperative day 3	1.65±0.73	1.52±0.66	*t* = 0.724	0.472
Postoperative day 7	1.95±0.86	1.76±0.92	*t* = 0.826	0.412
IgM, g/L				
At admission	1.98±0.84	1.87±0.77	*t* = 0.529	0.599
Postoperative day 3	1.42±0.76	1.02±0.55	*t* = 2.335	0.023
Postoperative day 7	1.83±0.96	1.52±0.85	*t =* 1.324	0.095
Complement C3, g/L				
At admission	1.07±0.25	1.08±0.19	*t* = 0.174	0.862
Postoperative day 3	1.34±0.34	1.22±0.32	*t* = 1.408	0.164
Postoperative day 7	1.22±0.27	1.13±0.16	*t* = 1.571	0.122
Complement C4, g/L				
At admission	0.30±0.19	0.28±0.12	*t* = 0.487	0.628
Postoperative day 3	0.38±0.15	0.33±0.17	*t* = 1.472	0.146
Postoperative day 7	0.25±0.13	0.27±0.11	*t* = 0.643	0.523

Data are reported as means±SD or number. ERAS: enhanced recovery after surgery.

The C3 and C4 levels initially increased and then decreased in both groups. These levels were generally higher in the ERAS group, but the difference was not statistically significant (P>0.05, Table 7).

### Cellular immunity

Following surgery, the number of total T lymphocytes (CD3^+^), helper T cells (CD3^+^CD4^+^), cytotoxic T cells (CD3^+^CD8^+^), and the CD4^+^/CD8^+^ ratio decreased initially and then increased in both groups. ERAS patients generally had higher total T lymphocyte counts, helper T cell counts, and CD4^+^/CD8^+^ ratios than the control patients. The differences between the two group were significant on postoperative day 3 in the case of the total T lymphocyte count (P<0.05), and on postoperative days 3 and 7 in the case of the helper T cell count and the CD4^+^/CD8^+^ ratio (P<0.05, Table 8).

**Table 8:** Comparison of T lymphocyte subsets between the two groups.

Item	ERAS group	Control group	Test value	P
Total T lymphocytes (CD3^+^), %				
At admission	71.32±16.71	73.58±18.25	*t* = 0.501	0.619
Postoperative day 3	55.56±15.72	46.81±17.43	*t* = 2.042	0.046
Postoperative day 7	69.58±20.72	61.96±22.45	*t* = 1.366	0.177
Helper T cells (CD3^+^CD4^+^), %				
At admission	45.28±14.62	46.86±17.30	*t =* 0.382	0.704
Postoperative day 3	33.72±12.31	27.19±11.14	*t* = 2.154	0.035
Postoperative day 7	42.89±14.67	35.04±15.55	*t* = 2.011	0.048
Cytotoxic T cells (CD3^+^CD8^+^), %				
At admission	26.87±12.71	27.11±10.85	*t* = 0.079	0.937
Postoperative day 3	23.78±8.12	21.23±8.68	*t* = 1.175	0.245
Postoperative day 7	27.29±11.52	25.38±12.64	*t* = 0.612	0.543
CD4^+^/CD8^+^ ratio				
At admission	1.63±0.45	1.74±0.83	*t* = 0.638	0.526
Postoperative day 3	1.40±0.35	1.19±0.42	*t* = 2.104	0.040
Postoperative day 7	1.57±0.40	1.37±0.36	*t* = 2.036	0.046

Data are reported as means±SD or number. ERAS: enhanced recovery after surgery.

## Discussion

This study showed that ERAS can provide significant clinical and economic benefits in gastric cancer patients undergoing radical gastrectomy. The time to first flatus and defecation after surgery, time to removal of drainage tubes, time to resumption of oral fluids and semi-liquid nutrition, and time to postoperative mobilization were all significantly shorter in the ERAS group than in the control group. The duration of postoperative infusion and hospitalization were also significantly shorter in the ERAS group. Hospitalization cost was lower in the ERAS group than in the control group, but the difference was not statistically significant. These findings are consistent with those of previous studies (16,17).

Serum albumin, which is synthesized in the liver, is the most abundant protein in the human body. It is involved in the transport of human metabolites, maintenance of plasma osmolality, nutrition, among other functions. Serum prealbumin is a transport protein with a short half-life of approximately 2 days. Its main function is to bind with and transport vitamins. It also enhances immunity by accelerating lymphocyte maturation. Serum albumin and prealbumin are sensitive markers of nutritional status in the perioperative period; they directly and rapidly reflect the changes in nutritional status in response to different measures (18). In the present study, serum albumin and prealbumin levels were significantly higher in the ERAS group than in the control group on postoperative day 7. The reason may be that ERAS patients had a faster transition to a liquid and semi-liquid diet.

While surgical resection is still the only option to cure gastric cancer, it can lead to a strong stress response. Stress inhibits immune function and stimulates the production of several inflammatory mediators (19), which further inhibit immune function. These changes result in increased postoperative complications, delayed recovery after surgery, and other adverse effects (20,21). CRP is an inflammatory protein synthesized and secreted by the liver in the acute phase of stress. It can activate complement, promote granulocyte proliferation, and enhance macrophage phagocytosis. Postoperative elevation in serum CRP level is closely related to the degree of surgical trauma (22). CRP can reflect acute stress and inflammatory response with high sensitivity and specificity. Some researchers have reported that CRP is an independent predictor of prognosis in gastric cancer patients (23). In this study, the mean CRP level in both groups was elevated on the first day after surgery, peaked on the third day, and then began to decline. Overall, the changes in the ERAS group were more moderate. The differences between the groups were statistically significant on postoperative days 3 and 7. The relatively lower CRP level in the study group suggests that ERAS can relieve stress and inflammatory response in gastric cancer patients during the perioperative period and may thus reduce the risk of postoperative infection.

Neutrophils are also sensitive indicators of the inflammatory response. In this study, the neutrophil count in both groups initially increased after surgery and then decreased. The count was always lower in the ERAS group than in the control group, with the difference being statistically significant on the third day after surgery. The differences in CRP level and neutrophil counts between the two groups suggest that the application of ERAS reduced the inflammatory response in gastric cancer patients (24).

Gastric cancer patients in China are mostly elderly and usually have advanced disease at presentation; most patients also have some degree of malnutrition. All these factors cause immunodeficiency (6). The onset, progression, and metastasis of tumors are associated with immune function. If ERAS can protect the immune function of cancer patients, it should be able to reduce the risk of recurrence and metastasis to a certain extent. We therefore examined the effects of ERAS on immune function in the perioperative period.

Both innate and specific immunity are involved in antitumor activity. Specific immunity plays the major role, with T lymphocytes, immunoglobulins, and complements all having important functions in the suppression of tumorigenesis (22). The main effector molecules are immunoglobulins, which are synthesized and secreted by activated human B lymphocytes or plasma cells. They mediate the humoral immune response by combining with the corresponding antigens. Human immunoglobulins mainly comprise five molecular types: IgA, IgD, IgE, IgM, and IgG. The role of IgD is not clear, and IgE secretion is unstable; therefore, IgA, IgM, and IgG are usually chosen to reflect humoral immune function in clinical studies. Immunoglobulin levels are associated with the resistance to infection, nutritional status, trauma, and other factors (25). In this study, postoperative immunoglobulin (IgA, IgM, and IgG) levels in both groups decreased initially and then rose, indicating that humoral immunity was inhibited by surgical trauma, anesthesia, and starvation. The postoperative immunoglobulin levels were always higher in the ERAS group than in the control group, and the changes more moderate in the ERAS group. The statistically significant differences between the groups in IgG and IgM on postoperative day 3 and in IgG on postoperative day 7, suggest that ERAS might reduce immunoglobulin consumption and accelerate immunoglobulin production, thereby facilitating the early recovery of humoral immunity after surgery.

Complements are protein molecules that have enzyme activity; they are widely involved in antibacterial and antiviral defense responses and in immune regulation. The two most important complements are C3 and C4. C3 is the convergence point of the classical and alternative activation pathways of the complement system. It has many biological fragments that can selectively act on different T lymphocyte subsets to regulate immune function. C4 is the complement activated next in the classical activation pathway; it is involved in immune identification and immune homeostasis (13,25). In the present study, the postoperative C3 and C4 levels tended to increase at first and then decrease in both groups. The levels were higher in the ERAS group than in the control group, but the differences between the groups were not statistically significant (P>0.05). The reason could be that complement has little effect on the changes produced by acute stress response and immune injury.

T lymphocytes derived from the bone marrow are the main cells involved in antitumor cellular immunity (26). T lymphocytes are highly heterogeneous, and can be divided into different subsets according to their surface markers and functional characteristics: helper T cells (CD3^+^CD4^+^), cytotoxic T cells (CD3^+^CD8^+^), and inhibitory Tcells. The number of lymphocytes in each subset fluctuates within a certain normal range in the healthy body, and they act in coordination with each other to maintain immune function. When the number of cells of a specific subset exceeds its normal range or its functions change, immune function is enhanced or inhibited. The CD4^+^/CD8^+^ ratio indirectly reflects the function of T lymphocyte subsets, and is positively correlated to cellular immune function. Studies have shown that surgical trauma, anesthesia, and other stresses impact the number and function of T lymphocytes, with the main manifestations being increased T lymphocyte apoptosis and decreased helper T cells and CD4^+^/CD8^+^ ratio (27). In the present study, the total T lymphocyte count, helper T cell count, and CD4^+^/CD8^+^ ratio decreased initially and then increased after surgery in both groups. The levels of all three parameters were higher in the ERAS group that in the control group on postoperative day 3. Helper T cell count and the CD4^+^/CD8^+^ ratio remained higher in the ERAS group on postoperative day 7; these differences were statistically significant.

These results suggest that compared to the control patients, ERAS patients sustained less damage to cellular immune function and showed faster recovery after surgery. Thus, ERAS may help reduce the consumption of T lymphocytes (especially helper T cells) and hasten the recovery of T lymphocyte function.
